# Indoor Air Pollution by Methylsiloxane in Household and Automobile Settings

**DOI:** 10.1371/journal.pone.0135509

**Published:** 2015-08-17

**Authors:** Fanyong Meng, Hao Wu

**Affiliations:** School of Economics and Management, Beihang University, Beijing, China; Tsinghua University, CHINA

## Abstract

This study examines characteristics of atmospheric methylsiloxane pollution in indoor settings where interior renovation/redecoration is being undertaken, in addition to ordinary family homes and inside family cars. Concentrations of atmospheric methylsiloxane in these locations were approximately one order of magnitude higher than that in outdoor areas. The average indoor concentration of methylsiloxane where renovation was being undertaken was 9.4 μg/m^3^, which is slightly higher than that in an ordinary family home (7.88 μg/m^3^), while samples from family cars showed lower concentration (3.10 μg/m^3^). The indoor atmospheric concentration during renovation/redecoration work was significantly positively correlated with the duration of the work. The structure of atmospheric methylsiloxane pollution is basically the same in these three venues. The concentration of annulus siloxane was much higher than that of linear compounds (85% of the total methylsiloxane concentrations). Household dust in average family homes showed total methylsiloxane concentration of 9.5 μg/m^3^ (average); the structure mainly consisted of linear siloxane (approximately 98% of total concentration), thereby differing from that of atmospheric methylsiloxane pollution. The comparatively high concentration of methylsiloxane in these three venues indicates that interior renovation and decoration work, and even travelling in cars, can involve exposure to more serious siloxane contamination during everyday activities.

## Introduction

Methylsiloxane is widely used in a variety of interior redecoration materials, furniture, daily consumables, and car accessories, such as polishing agents, paints and varnishes, sealants, textiles, personal care products, and lubricants, etc. [1–2]. With the use of these products, large quantities of methylsiloxane enter the surrounding environs through various emission pathways or as a result of leakage. Methylsiloxane pollution has been reported in water [3], soil [4], and in sediments and organisms [2, 5–7]. However, few studies have examined pollution in the general atmosphere in ordinary indoor environs. Methylsiloxane is relatively stable, and is therefore common in the environment. In recent years, reports have indicated that some methylsiloxane substances are potential endocrine disruptors [8] and show neurotoxic effects in addition to reproductive toxicity [2]. Consequently, the impact of methylsiloxane pollutants on human health and the environment is an issue of increasing concern.

Since construction and redecoration products and daily household products containing high concentrations of methylsiloxane additives are widely used in everyday life, this study specifically examines atmospheric concentrations in homes undergoing renovation work, in homes that have been occupied for some time, and in private cars.

## Materials and Methodology

### Sample Collection

Field samples were obtained following voluntary participation in the study by householders and owners of private cars. The China Recycle Economy Research Center of Beihang University provided necessary support for the collection of all samples in this study. All participants volunteered permission to conduct the study in their homes/cars; the field studies did not involve endangered or protected species, and therefore no specific permissions were required for the sample collection. Samples were collected in August 2014 in the Beijing area. Air samples were collected with 500 mg ENV+ cartridges, which were connected to a diaphragm pump with Teflon tubing. The pump was used to pull air through the cartridges for 24 hours at a flow rate of 0.5 L/min. Indoor air quality samples were collected from 40 family homes while redecoration work was underway, from 30 family homes that the families had occupied for more than two years, and from 20 private cars. Indoor temperatures of the sampling sites were approximately 26–30°C. In addition, 10 household dust samples were obtained from homes that were occupied for more than two years, and 5 outdoor air samples were collected from sites near the occupied residential buildings (those that were occupied for more than two years and not influenced by redecoration work). The time schedules for the home redecoration work, as well as the different moving-in dates were also collated; for example, in the homes being renovated, the work schedules were measured accordingly (10–60 days), while for those homes that residents had occupied for some time, the exact date of occupancy and the permanent members of the household were collated.

### Standards and Reagents

Annular siloxane standards (D4, D5, D6, purity> 98%), linear siloxane standards [L4, L5 and dimethyl siloxane mixture standards (PDMS, L5-L16), purity> 98%)], and the internal standard (trimethylsilyoxy) M4Q (purity 97%) were purchased from Sigma-Aldrich Corporation. Dichloromethane, hexane, and acetone were purchased from Fisher Scientific (USA). Prior to use, anhydrous sodium sulfate and chromatographed on silica gel were dried in a muffle furnace at 450°C for 4 hours.

### Sample Pretreatment

After the atmospheric samples were collected, an ENV + solid-phase extraction column was eluted with n-hexane solvent. The eluent was then concentrated into the GC/MS for quantitative detection. The dust samples were extracted using n-hexane/ethyl acetate solvent; the extract was then collected and concentrated with nitrogen, and finally used for quantitative detection.

### Sample Preparation and Quality Control (QA/QC)

Strict quality control measures were adopted during sample collection, preservation, pretreatment, and analysis in order to minimize background contamination: the process for acquiring atmospheric samples eliminated the use of any products containing silicone ingredients; sampling and pretreatment operators were prohibited from using any personal care products; the sampling process also collected blanks in the same field; GC/MS analysis was conducted under the premise of meeting the test requirement, and lower inlet temperature and lower loss of the capillary column were chosen whenever possible.

Despite adopting the above measures, the blank samples in the field still showed detectable concentrations of annular siloxane D4–D6. The concentrations of the three types of annular methylsiloxane in the blank atmospheric samples were 0.22–0.36 ng/m^3^, while those in the blank dust samples were 0.17–0.89 ng/g. Therefore, the concentrations detected in the blank samples should be deducted from the corresponding results for the actual samples tested in the laboratory. The Limit of Quantitation (LOQ) of the 16 methylsiloxanes in atmospheric and dust samples was 0.17–1.8ng/m^3^ and 0.5–2.4 ng/g respectively.

## Results and Discussion

### Characteristics of Indoor Methylsiloxane Pollution during Home Redecoration

**Fig 1:**
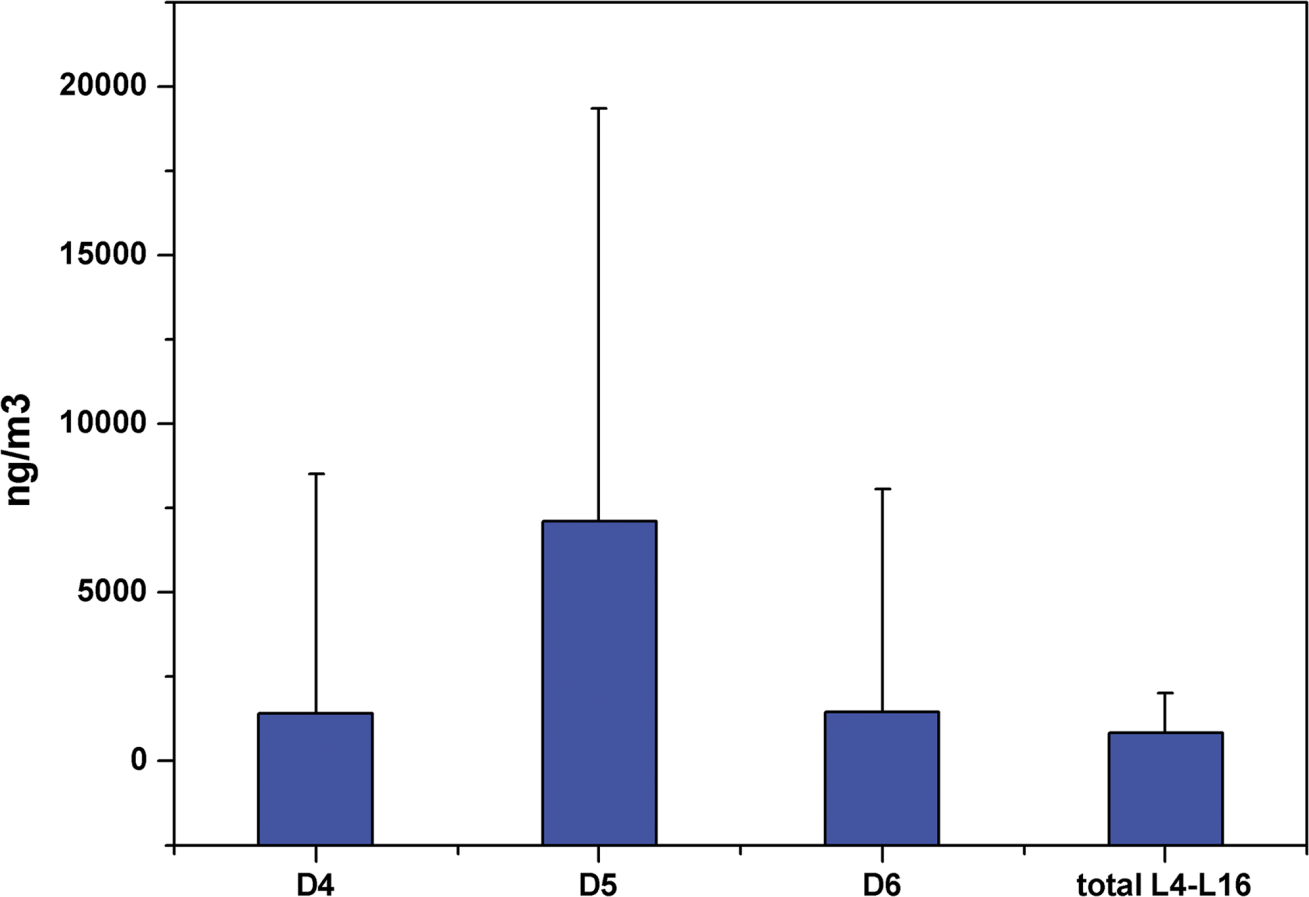
Concentrations of Methylsiloxanes in Indoor Atmosphere in Homes Undergoing Redecoration.

This study examined all atmospheric methylsiloxanes (D4–D6 and L4–L16) in 40 homes where redecoration work was underway. D4–D6 concentrations were within the ranges LOQ–13.5 (average concentration 1.5), 0.08–156 (6.7), and 0.001–8.3 μg/m^3^ (1.7 μg/m^3^), respectively. Total annular methylsiloxane concentration was 0.20–169 μg/m^3^ (average total concentration 9.6 μg/m^3^), which was correlated with the usage volume of redecoration materials containing methylsiloxanes. Products/additives show higher concentrations of linear methylsiloxanes, but these species have lower volatility than annular methylsiloxane. Total concentrations of linear (methylsiloxane) compounds L4–L16 detected in the atmosphere were between 2.3 ng/m^3^ and 3.4 μg/m^3^ (detection range 25–97%), which were lower than those for annular methylsiloxanes (Fig 1, S1 Table). The total concentration of methylsiloxanes varied within a wider range of 0.20–172 μg/m^3^ (average concentration 2.6 ± 13.1 μg/m^3^). Analysis of the time schedules for renovation work showed that redecoration duration (days) was significantly positively correlated with indoor atmospheric concentrations of methylsiloxanes (p<0.05, r = 0.65). Although volatile methylsiloxanes contained in redecoration materials can constantly be dispersed in outdoor environments, with the intensification of the redecoration processes, more materials containing siloxanes were delivered to the site, resulting in gradually increasing indoor concentrations of methylsiloxanes within homes undergoing renovation/redecoration.

### Characteristics of Indoor Atmospheric and Dust Methylsiloxane Pollution in Ordinary Family Homes

Atmospheric methylsiloxanes (D4–D6 and L4–L16) were also detected in the 30 ordinary family homes not undergoing renovation. The concentrations of D4, D5, and D6 were within the ranges <LOQ–1.93 (average concentration 0.39), 0.08–20.8 (5.15), and 0.001–2.36 μg/m^3^) (0.74 μg/m^3^) respectively. The total concentrations of the three types of annular methylsiloxanes ranged from 0.09 to 23.0 μg/m^3^ (average concentration 6.27 μg/m^3^). Total concentration of linear (methylsiloxane) compounds L4–L16 ranged from 0.008 to 6.77 μg/m^3^ (total average concentration 0.74 μg/m^3^).

**Fig 2:**
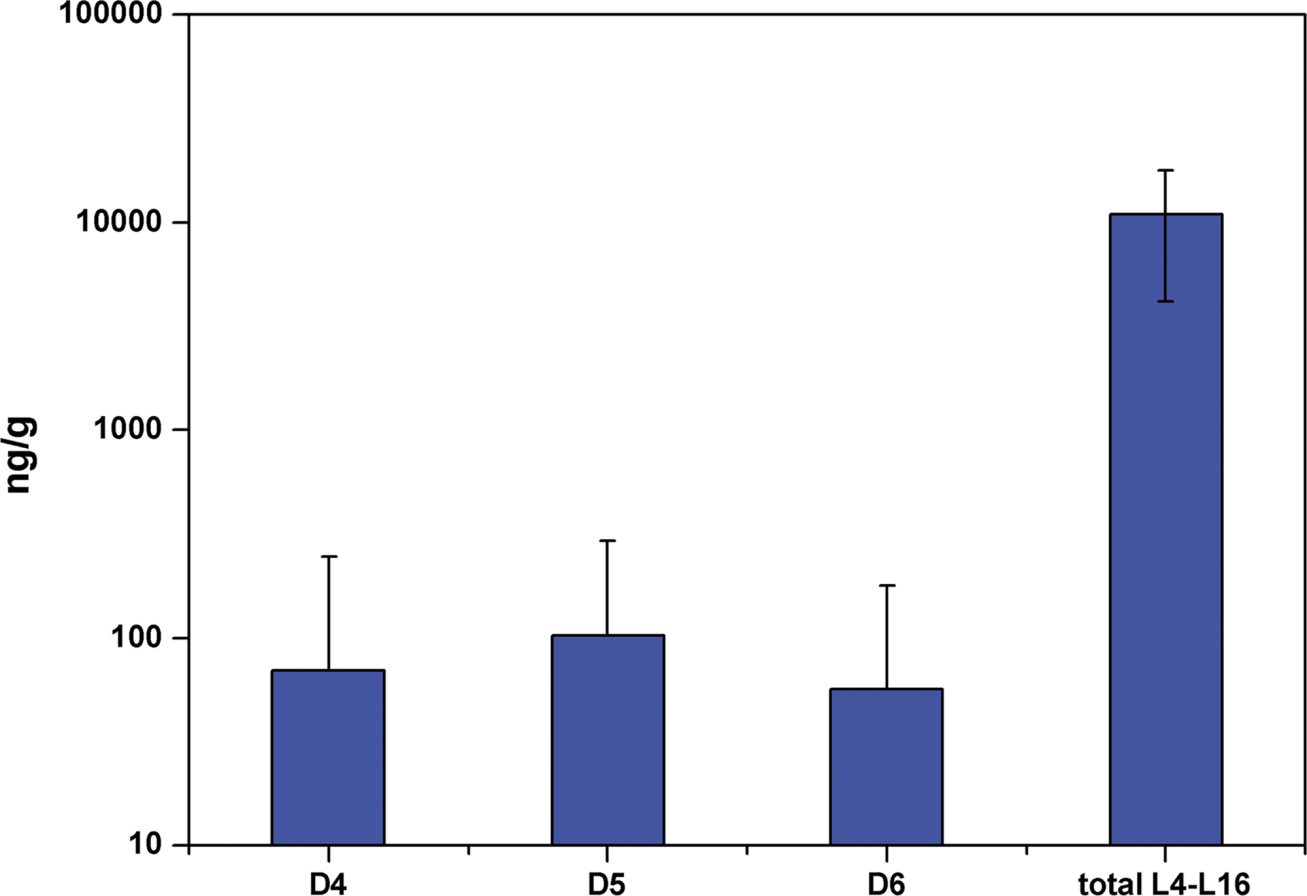
Concentrations of Methylsiloxanes in Dust in Ordinary Family Homes.

Ten dust samples were also collected from the 30 ordinary family homes at the same time. Total methylsiloxane concentrations in the dust ranged from 2.44 to 24.6 μg/g (average 10.3 ug/g). Concentrations of annular methylsiloxanes D4–D6 were within the ranges 0.40 (0.07), <LOQ–0.34 (0.09), and <LOQ–0.24 μg/g (0.04 μg/g), respectively. The total concentrations of linear siloxanes (L4–L16) ranged from 2.33 to 24.5 μg/g (average 10.1 μg/g) (Fig 2, S2 Table), representing approximately 98% of total methylsiloxane concentration. The distribution characteristics of methylsiloxane pollution in dust differ entirely from those in the atmosphere, which is consistent with previous reports [9–10]. Analysis was carried out to establish the reason for this variation, which might be due to the weak volatility of the linear siloxanes compared with annular siloxanes. With the result that linear siloxane species were more prevalent in dust, whereas annular siloxanes mainly existed in gaseous phase. Despite the limited number of dust samples collected, it was found that the concentration of methylsiloxanes in dust was positively correlated with that in the gaseous phase (p<0.05).

### Characteristics of Atmospheric Methylsiloxane Pollution inside Family Cars

In the 20 samples obtained from private cars, total atmospheric concentrations of annular (D4–D6) and linear siloxanes (L4–L16) were within the ranges 0.2–5.3 (1.73) and 0.04–1.79 μg/m^3^ (0.51 μg/m^3^) respectively. Annular siloxane accounted for 77% of total methyl siloxane concentration, which is slightly lower than in the two types of indoor atmospheric samples (94% and 89%), probably because the additive form of methylsiloxane within materials used in automotive interior/products or consumables is slightly different from that in interior redecoration and household products. The age of the car was not significantly correlated with the atmospheric concentration of methylsiloxane within the vehicle (p> 0.05).

### Comparisons of Three Indoor Settings and the Target in Outdoor Atmosphere

**Fig 3:**
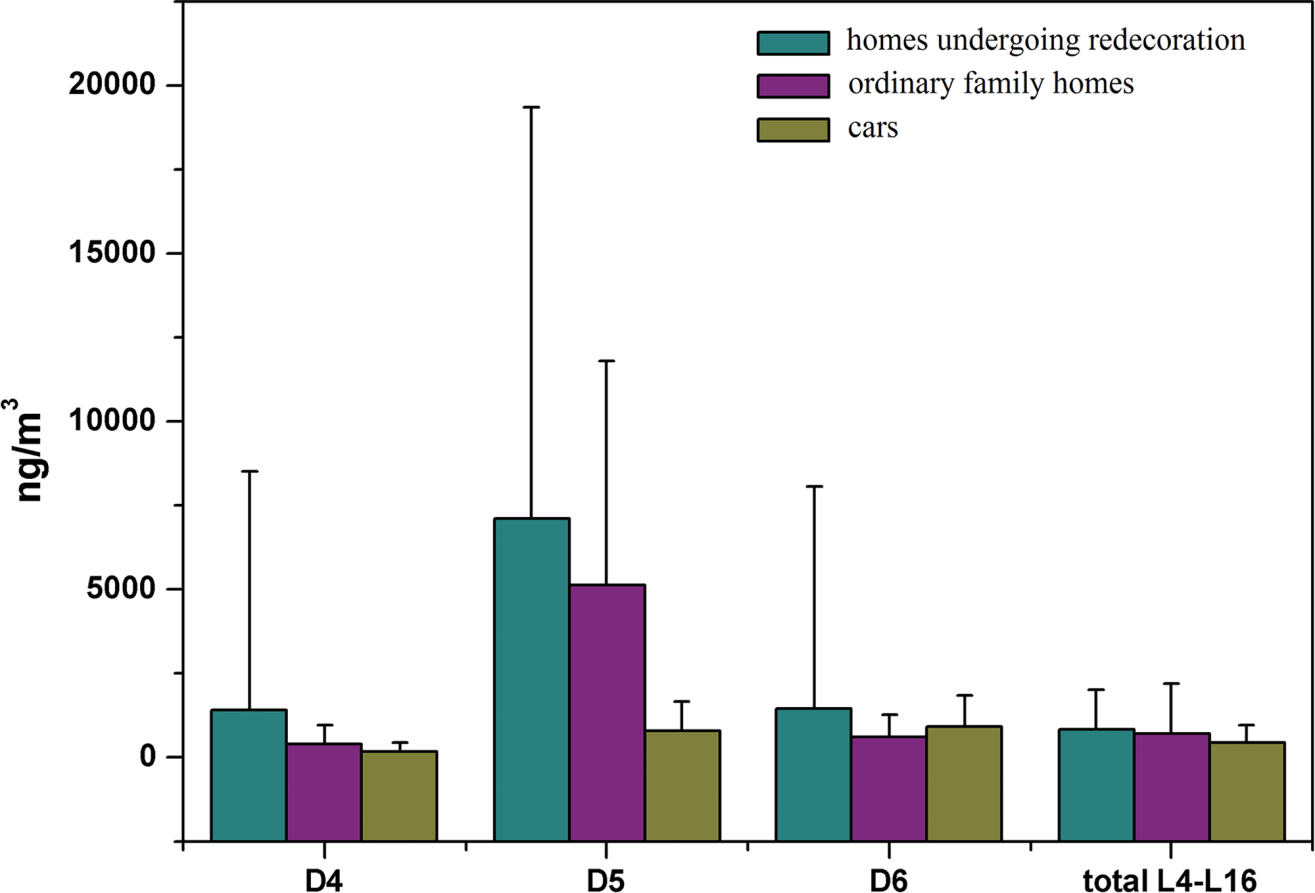
Comparisons of Atmospheric Methylsiloxane Concentrations in Three Indoor Settings.

Fig 3 shows that the indoor atmospheric concentration of annular methylsiloxane during interior redecoration is slightly higher than that within the ordinary family homes and the vehicles(S1, S3 and S4 Tables). Furthermore, the atmospheric concentration of D5 inside cars is significantly lower than in indoor atmosphere. The methylsiloxanes in rooms being redecorated originate from various redecoration materials/products, whereas those in ordinary family homes originate from furniture, household electrical goods, personal care products, and various kinds of consumables. It can be seen that interior redecoration can result in higher levels of methylsiloxane pollution. This is likely due to the usage volume of redecoration materials, such as paint, latex paint, etc. Many methylsiloxanes in redecoration materials enter the indoor atmosphere. Fewer methylsiloxane-containing materials are used in ordinary family homes compared with homes undergoing interior renovation. Higher concentrations of atmospheric methylsiloxanes were detected inside cars, which likely derive from multiple vehicle components such as car seats, interior fixtures and fittings, as well as automotive oils and lubricants, which together represent sources of serious methylsiloxane pollution. No linear siloxane species were detected in the five outdoor atmospheric samples, whereas the concentrations of annular methylsiloxanes D4, D5, and D6 ranged from <LOQ to 37.1 ng/m^3^, approximately one magnitude lower than those in the three indoor settings.

To date, there are few studies on methylsiloxane pollution in indoor atmosphere. Shields et al. [11] collected 70 indoor atmospheric samples from rooms within the US Department of Commerce and detected D4 and D5 in 69 of the samples (2.5–10 and 7.0–39.6 μg/m^3^ respectively). In Sweden [12], atmospheric samples were taken from 400 family homes, which showed average D4, D5, and D6 concentrations of 9.7, 9.7, and, 7.9 μg/m^3^, respectively. Both of those studies reported higher concentrations of methylsiloxanes than those detected in the present study, in either the homes undergoing redecoration or the ordinary family homes. Lu et al. [13] tested for annular methylsiloxanes (D4–D6) in personal care products available on the Chinese market, and found that average methylsiloxane concentrations were 5.24, 29.7, and 18.3 μg/g. Horii et al. [14] reported that the average concentrations of the three compounds in personal care products on the US market were 141, 2890, and 896 μg/g, respectively. Those concentrations are far higher than were found in products available on the Chinese market. This perhaps explains why the atmospheric methylsiloxane concentrations detected inside Chinese family homes are lower than those detected in the USA and Sweden. We also tested industrial additives/products (paints, varnishes and polishes), which showed methylsiloxane concentrations that were similar or even lower than those in the personal care products reported in the USA. This may explain why the atmospheric methylsiloxane concentrations in rooms undergoing redecoration work in this study are still slightly lower than those reported for family homes in other countries.

## Conclusion

Concentrations of atmospheric methylsiloxane in indoor homes undergoing interior renovation/redecoration were higher than in ordinary family homes, while samples obtained from family cars showed lower concentrations. Overall, the higher concentrations of methylsiloxane in the above three venues were higher than those in outdoor venues, indicating that interior renovation and decoration work, and even traveling in cars, can involve exposure to more serious siloxane contamination during everyday activities.

## Supporting Information

S1 Table Concentrations of methylsiloxanes in indoor atmosphere in homes undergoing redecoration. (XLS)

S2 Table Concentrations of methylsiloxanes in dust in ordinary family homes. (XLS)

S3 Table Concentrations of methylsiloxanes in indoor atmosphere in ordinary family homes. (XLS)

S4 Table Concentrations of methylsiloxanes in indoor atmosphere in cars. (XLS)
